# Analysis of factors affecting crash under risk scenarios based on driver homogenous clustering

**DOI:** 10.1371/journal.pone.0293307

**Published:** 2023-10-20

**Authors:** Lili Zheng, Yanlin Li, Tongqiang Ding, Fanyun Meng, Yanlin Zhang

**Affiliations:** 1 School of Transportation, Jilin University, Changchun, China; 2 Jilin Research Center for Intelligent Transportation System, Changchun, China; 3 Jilin Province Key Laboratory of Road Traffic, Changchun, China; Texas A&M Transportation Institute, UNITED STATES

## Abstract

Research on road safety has focused on analyzing the factors that affect crashes. However, previous studies have often neglected differences in crash causation among heterogeneous clusters of drivers. In particular, the differences in the combined effect mechanisms of the factors in the risk scenarios have not been completely explained. Therefore, this study used the K-means algorithm to perform multidimensional feature homogeneous clustering for drivers involved in crashes and near-crashes. Structural equation modeling involving mediating effects was introduced to explore the direct and indirect effects of each influencing factor on vehicle crashes under risk scenarios and compare the differences in crash causation among different driver clusters. The results indicate that the drivers who experienced the risk scenarios can be classified into two homogeneous driver clusters. Significant differences exist in the demographic characteristics, intrinsic driving characteristics, and crash rates between them. In the risk scenario, traffic factors, distraction state, crash avoidance reaction, and maneuver judgment directly affect the crash outcomes of the two cluster drivers. Demographic characteristics and environmental factors have fewer direct influence on the crash outcomes of two-cluster drivers, but produce more complex mediating effects. Analysis of the differences in the influence of factors between clusters indicates that the fundamental cause of crashes for cluster 1 drivers includes poor driving skills. In contrast, cluster 2 drivers’ crashes were more influenced by traffic conditions and their safety awareness. The analysis method of this study can be used to develop more targeted road safety policies to reduce the occurrence of vehicle crashes.

## 1. Introduction

Traffic accidents have long caused severe personal injuries and financial losses. Each year, the lives of approximately 1.3 million people worldwide are terminated by road traffic accidents, and another 20–50 million people suffer non-fatal injuries, with traffic accidents accounting for 3% of the gross domestic product of most countries [1]. Traffic accidents have caused tremendous financial loss and painful emotional damage to countless families. Road safety researchers have conducted multifaceted studies to improve traffic safety. One of the most critical research directions involves determining the influential factors and mechanisms of crashes. Knowledge of crash causes is essential because it directs the mind to consider potential preventive actions [2].

Existing studies have conducted in-depth analyses of the factors that influence crash events. Lyon et al. [3] used multivariate logistic regression modeling to explore the effects of driver age on crash events. The results indicated that young and older drivers were more likely to experience crash events than middle-aged drivers. Hao et al. [4] used the support vector machine (SVM) method to investigate the causes of single-vehicle crashes in naturalistic driving datasets. The sensitivity analysis results of the SVM classifier confirmed that the risk-driving behavior was the most significant influencing factor. Yu et al. [5] used a random forest algorithm to model emergency braking behavior. The results show a direct relationship between traffic density, road conditions, and the occurrence of crash events. In addition to the direct effects of each influencing factor on crash events, they commonly have complex indirect effects. For example, drivers in a relatively stable traffic environment are vulnerable to distractions, which can lead to crash events [6]. Gender is directly related to crash events and indirectly affects crash events through security attitudes [7]. Male drivers tend to ignore or even resist in-car warning messages, whereas female drivers are more likely to correct their errors. The above literature shows that crash events are primarily influenced by drivers’ demographic characteristics, driving performance, environmental factors, and traffic factors.

Although current research on the factors influencing crash events has been productive, two limitations still exist in the related studies. First, previous studies have tended to target all drivers, and few studies have further analyzed the variability in the effects of influencing factors among different driving clusters. In fact, some drivers are prone to crash events and have a higher probability of repeating them after their occurrence, and this cluster of drivers can be considered a high-risk driver cluster. Different clusters of drivers not only differ in the frequency of accidents but also in the effect of factors influencing vehicle crashes. The different subjects in the study may lead to contradictory conclusions about the effect of the same factor on crash events. Therefore, to accurately investigate the mechanism of the effect of crash event-influencing factors, it is necessary to first classify the cluster of drivers.

For driver cluster classification, gender, age, and crash and near-crash (CNC) rates were the most common indicators. Wang and Xu [8] classified drivers into three clusters based on CNC rates using the K-means algorithm, and indicated that the high CNC rate cluster was more likely to experience distracted behavior. Michaels, Chaumillon [9] empirically classified drivers between the ages of 16 and 84 into three different age clusters: older, middle-aged, and young. The study showed that middle-aged and older drivers were more likely to be involved in fault crash events than young drivers. Other studies have directly divided the driver clusters by gender, indicating that female drivers significantly reduce risky driving behavior more than male drivers as their driving experience increases [10]. Some studies have also collected intrinsic characteristics of drivers in the form of questionnaires to classify driving clusters based on differences in intrinsic characteristics. Yang et al. [11] designed a questionnaire consisting of three subscales to investigate drivers’ driving skills, driving behaviors, and driving aggression, and used the K-means algorithm to classify drivers into different clusters using their behavioral aggression and emotional aggression as input features. With the development of sensor devices, research methods for collecting vehicle parameters, analyzing driver operation characteristics, and classifying clusters based on operation characteristics are becoming increasingly common. Das and Ahmed [12] used the speed, acceleration, and yaw rate as clustering input features to classify drivers into two clusters: conservative and aggressive. The results indicate that drivers in the conservative cluster take longer to change lanes during foggy weather, which may reduce the probability of accidents. As seen from the above literature, scholars have usually divided driver clusters based on a single characteristic. However, human factors associated with crash events often include demographic characteristics, driving style, operational skills, and safety awareness. A single feature cluster classification can only differentiate driver clusters from a certain perspective; however, it does not reflect the cluster differences from the perspective of the crash cause and does not meet the requirements of homogeneous driver cluster clustering.

Another limitation of the research on influencing factors is that previous articles usually used security events and CNC data to develop comparative impact factor studies and discuss how to prevent CNCs. However, from the perspective of reducing injuries and minimizing financial losses, we are more concerned with avoiding crashes in risk scenarios.

Several studies have identified that young drivers cannot detect the occurrence of risk scenarios in time owing to inexperienced driving, which leads to car accidents [13–15]. Female drivers have a lower level of vehicle handling than male drivers and are more likely to react incorrectly to crash avoidance in risky situations, leading to crashes [16, 17]. The larger braking distances required when the driver is at an excessive speed increase the likelihood of crash events [18]. Complex intersections [17] and particular road alignments [19] reduce driver options for crash avoidance strategies and can easily lead to crashes. The aforementioned studies have provided multiple perspectives on the causation of event outcomes. However, they have not been able to clearly explain the combined effects of multiple influencing factors in risk scenarios and much less analyze the differences in the causation of crashes between heterogeneous clusters of drivers. Clarifying the mechanisms of influencing factors in risk scenarios is crucial for developing risk prevention policies, eliminating potential risk factors, and avoiding crashes.

Beyond the aforementioned issues, there are certain shortcomings in the quantification methods applied to assess the effects of influencing factors in existing studies. These studies predominantly rely on regression techniques, with logistic regression being a prime example [20, 21]. However, it’s crucial to recognize that traditional regression methods have certain limitations when applied to the domain of traffic safety analysis. Firstly, logistic regression cannot quantify the influence of latent variables through the evaluation of observed variables. Moreover, this method allows variables to be either dependent or independent in one analysis, making it unable to capture the mediating effects of certain influencing factors. However, Structural equation modeling (SEM) effectively addresses the issues mentioned above. It integrates factor analysis (measurement model) and structural relationships (structural model) into a cohesive framework. Furthermore, SEM permits influencing factors within the structural equation to serve dual roles as both dependent and independent variables. This facilitates the quantification of intricate relationships between observed and latent variables, as well as the assessment of mediating effects of influencing factors [22]. In addition to the advantages mentioned above, SEM also has the capability to compute measurement errors of independent variables and assess the impact of multicollinearity. Due to its unique strengths in analyzing factor effects, SEM has become increasingly prevalent in the field of traffic safety in recent years [23–25].

In summary, there are two main shortcomings in existing collision influencing factor analysis research: (1) Existing studies have typically not categorized homogeneous clusters of drivers based on accident causation when developing influencing factor analysis models, and as a result, they can not provide individualized influencing factor analysis results. (2) Current research often lacks a clear explanation of the cumulative effects of the numerous influencing factors in risk scenarios. In response to these issues, this study aims to achieve the following objectives:

- The method of multidimensional feature clustering of drivers was used to comprehensively classify homogeneous clusters of drivers with different risk levels from three aspects: demographic characteristics, driving characteristics, and crash rates.
- SEM with mediating effects was introduced to comprehensively consider the combined effects of demographic characteristics, driving performance, traffic factors, and environmental factors on crash outcomes under risk scenarios.
- Comparing the variability in the effects of influencing factors across driver clusters allows the results of this study to suggest more targeted safety countermeasures for drivers and provide a theoretical basis for developing road safety intervention policies.

The remaining sections of this study are organized as follows: Section two presents the basic information of the data, along with statistical and coding results. Section three outlines the methods for driver clustering and crash influencing factor analysis. Section four provides the results of driver cluster segmentation and quantifies the relationships among various factors. Section five discusses the analysis of the research findings. Section six concludes this study by summarizing the main findings, identifying limitations, and suggesting directions for future research.

## 2. Data

### 2.1 Data description

Data from a 100-car naturalistic driving study were used for this study. The 100-car study is the first large-scale naturalistic driving data collection study conducted in the U.S. The study sites were located in northern Virginia and Washington, DC. The study involved 102 drivers and 100 data collection vehicles, with a total of 2 million vehicle miles and 43,000 h of driving data collected over 12 consecutive months of naturalistic driving data [26]. In the 100-car study, advanced data acquisition devices were installed on vehicles, which classified naturalistic driving events into three types based on vehicle kinematic information and video records: safety, crash, and near-crash. A crash is an event in which a crash occurs between the subject vehicle and another vehicle, a fixed object, a pedestrian, a rider, or an animal. A near crash is an event in which a driver avoids a crash by a quick evasive maneuver when a crash occurs. The maneuver includes steering, braking, acceleration, and any combination of the above actions.

To make the results of this study reliable and explainable, all the data with missing information were deleted, and the uncertain description data (other, no analyzed data) with less than 2% were removed. 96 drivers with CNC records, 68 crash records, and 760 near-crash records were extracted.

### 2.2 Data processing

#### 2.2.1 Clustering data statistics

Before cluster segmentation, summary statistics were required for the personal and driving record data of the 96 drivers. **Table 1** presents the descriptive statistical information for the 96 drivers. **Table 2** presents the percentages of each feature subcategory in each driver’s CNC data. In **Table 2**, the crash ratio refers to the proportion of the number of crash events for a driver to the total number of CNC events for that driver; the maneuver judgment describes whether the driver’s driving behavior is safe and legal during the risk event, for example, it is unsafe and illegal for a driver to overtake across a solid lane line; Event nature has three types, which are single vehicle event, straight line event and steering event, where single vehicle event refers to the event where the driver is at risk of crashing with roadblocks, animals, etc; Pre-incident maneuver describes whether the driver has accelerated or steered prior to the risk event; Driver reaction refers to the driver’s crash avoidance operation for the risk scenario, which contains explicitly four types of braking, steering, no reaction, and acceleration; Distraction state refers to the severity of a driver’s distraction when a risk scenario occurs, and distractions can be classified into four risk levels based on the impact of various distractions on the outcome of the risk event [20]; Multiple distractions event ratio is the percentage of events where the driver has multiple distractions in the risk scenario. The formulae for each part of **Table 2** are as follows.

**Table 1:** Driver descriptive statistics.

Variables	Description	Count	Percentage
**Gender**	Female: 0	38	39.58%
	Male: 1	58	60.42%
**Age**	18–22: 0	19	19.79%
	23–27: 1	21	21.88%
	28–32: 2	10	10.42%
	33–37: 3	6	6.25%
	38–42: 4	8	8.33%
	43–47: 5	6	6.25%
	48–52: 6	10	10.41%
	53–57: 7	11	11.46%
	Over 58: 8	5	5.21%

**Table 2:** Driving characteristic descriptive statistics.

Variables	Min	Max	Mean	SD
**Crash ratio**	0.0%	100.0%	11.8%	22.7%
**Maneuver judgment**				
Safe and legal event ratio	0.0%	100.0%	88.8%	18.8%
Safe but illegal event ratio	0.0%	20.0%	1.0%	3.3%
Unsafe but legal event ratio	0.0%	50.0%	4.1%	9.0%
Unsafe and illegal event ratio	0.0%	100.0%	6.1%	16.2%
**Event nature**				
Single-vehicle event ratio	0.0%	66.7%	10.1%	15.9%
Straight-line event ratio	0.0%	100.0%	62.6%	28.1%
Steering event ratio	0.0%	100.0%	27.3%	27.1%
**Pre-incident maneuver**				
Steady driving event ratio	0.0%	100.0%	39.3%	28.6%
Direction change event ratio	0.0%	75.0%	17.3%	17.6%
Velocity change event ratio	0.0%	100.0%	43.4%	26.3%
**Driver reaction**				
Braking event ratio	0.0%	100.0%	82.1%	22.4%
Steering reaction event ratio	0.0%	75.0%	7.6%	13.7%
Accelerated event ratio	0.0%	33.3%	1.0%	4.2%
No reaction event ratio	0.0%	100.0%	9.3%	19.9%
**Distraction state**				
No-risk distraction event ratio	0.0%	100.0%	58.1%	29.0%
Low-risk distraction event ratio	0.0%	100.0%	31.9%	28.3%
Mid-risk distraction event ratio	0.0%	100.0%	8.0%	15.4%
High-risk distraction event ratio	0.0%	33.3%	2.0%	6.4%
**Multiple distractions event ratio**	0.0%	75.0%	14.8%	18.2%


$$P_{i}^{j}=\frac{N_{i}^{j}}{N_{i}^{CNC}}$$ (1)

$$P_{\min}^{j}\leq P_{i}^{j}\leq P_{\max}^{j},i\in \{1,\ldots ,n\}$$ (2)

$$P_{mean}^{j}=\frac{\sum_{i=1}^{n}P_{i}^{j}}{n}$$ (3)

Where $P_{i}^{j}$ is the proportion of events of characteristic subclass *j* for the driver *i*, $N_{i}^{j}$ is the number of events of characteristic subclass *j* for the driver *i*, and $N_{i}^{CNC}$ is the total number of CNC events for the driver *i*. $P_{\min}^{j}$, $P_{\max}^{j}$, and $P_{mean}^{j}$ are the minimum, maximum, and average occurrence rates of characteristic subclass *j* events, respectively. In this study, *i* is 96 and *j* has 20 categories.

#### 2.2.2 Structural equation data coding

To perform structural equation modeling operations, variables in the CNC data were first encoded. Sequential variables were coded according to their numerical size, and the remaining variables were dichotomized. The dichotomous method in SEM can efficiently measure the nonlinear effects of categorical variables on endogenous variables [27]. **Table 3** presents the results of the statistical description of the CNC data.

**Table 3:** CNC data descriptive statistics.

Variables	Description	Count	Percentage
**Crash outcome**	Near-crash: 0	760	91.80%
	Crash: 1	68	8.20%
**Gender**	Female: 0	388	46.86%
	Male: 1	440	53.14%
**Age**	18–22: 0	291	35.14%
	23–27: 1	140	16.91%
	28–32: 2	65	7.85%
	33–37: 3	26	3.14%
	38–42: 4	109	13.16%
	43–47: 5	58	7.00%
	48–52: 6	48	5.80%
	53–57: 7	36	4.36%
	Over 58: 8	55	6.64%
**Driver reaction**	Braking: 0	673	81.28%
	Others: 1	155	18.72%
**Maneuver judgment**	Safe: 0	730	88.16%
	Unsafe: 1	98	11.84%
**Distracting behavior 1**	No-risk distraction: 0	605	73.07%
	Low-risk distraction: 1	114	13.77%
	Mid-risk distraction: 2	64	7.73%
	High-risk distraction: 3	45	5.43%
**Distracting behavior 2**	No-risk distraction: 0	758	91.55%
	Low-risk distraction: 1	35	4.23%
	Mid-risk distraction: 2	17	2.05%
	High-risk distraction: 3	18	2.17%
**Multiple distractions**	No: 0	728	87.92%
	Yes: 1	100	12.08%
**Weather**	Clear: 0	638	77.05%
	Others: 1	190	22.95%
**Lighting**	Daylight: 0	538	64.98%
	Others: 1	290	35.02%
**Relation to junction**	Non-junction: 0	475	57.37%
	Others: 1	353	42.63%
**Locality**	Interstate and open country: 0	360	43.48%
	Others: 1	468	56.52%
**Traffic control**	No: 0	227	27.42%
	Yes: 1	601	72.58%
**Traffic density**	Level-of-service A: 0	220	26.57%
	Level-of-service B: 1	297	35.87%
	Level-of-service C: 2	213	25.72%
	Level-of-service D: 3	69	8.33%
	Level-of-service E: 4	25	3.03%
	Level-of-service F: 5	4	0.48%

## 3. Methods

### 3.1 K-means clustering model

First, a clustering algorithm was used to classify clusters of drivers with different risk levels. K-means is a division-based clustering algorithm widely used in various fields. The Euclidean distance is commonly used as an index to measure the similarity between data objects. Similarity is inversely proportional to the distance between data objects; the greater the similarity, the smaller is the distance. It has the characteristics of modest computation, rapid and effective processing of large datasets, quick convergence speed, and an excellent clustering effect. Moreover, previous studies have demonstrated that the clustering performance of the K-means clustering algorithm is relatively strong when dealing with multiattribute data [28]. Based on the above characteristics, this study used the K-means clustering algorithm with the descriptive statistics in **Tables 1 and 2** as input characteristics for the driver clustering study.

When applying the K-means clustering algorithm, determining the number of clusters is crucial for determining excellent or lousy classification performance. This study determines the optimal number of clusters by silhouette coefficient (SC) method.

### 3.2 Structural equation model

After classifying driver clusters, this study used structural equation modeling to assess the effects of the influencing factors. SEM is a widely used multivariate statistical analysis method that can be used to study the relationship between latent and observed variables, and the relationship between latent and latent variables. Compared with traditional linear regression methods, it can efficiently explain causal and mediating effect associations between variables [27].

Generally, the structural equation includes measurement and structural models. The measurement model characterizes the latent variables in terms of the corresponding observed variables. Its formula is expressed as follows:

$$X=\Lambda_{x}\xi +\delta$$ (4)

$$Y=\Lambda_{y}\eta +\varepsilon$$ (5)

Where *X* denotes the vector of observed exogenous variables; Λ_*x*_ denotes the matrix of structural coefficients for latent exogenous variables to their observed indicator variables; *ξ* denotes the vector of latent exogenous variables; *δ* denotes the vector of measurement error terms for observed variables. *Y* denotes the vector of observed endogenous variables; Λ_*y*_ denotes the matrix of structural coefficients for latent endogenous variables to their observed indicator variables; *η* denotes the vector of latent exogenous variables; *ε* denotes the vector of measurement error terms for observed endogenous variables.

A structural model is employed to elucidate the relationships between various latent variables, and it is formulated as follows:

η=Bη+Γξ+ζ (6)

Where *B* denotes the matrix of structural coefficients between endogenous latent variables; Γ denotes the matrix of structural coefficients for exogenous latent variables to endogenous latent variables; *ζ* denotes the unexplainable part of latent variables contained in the model.

**Fig 1:**
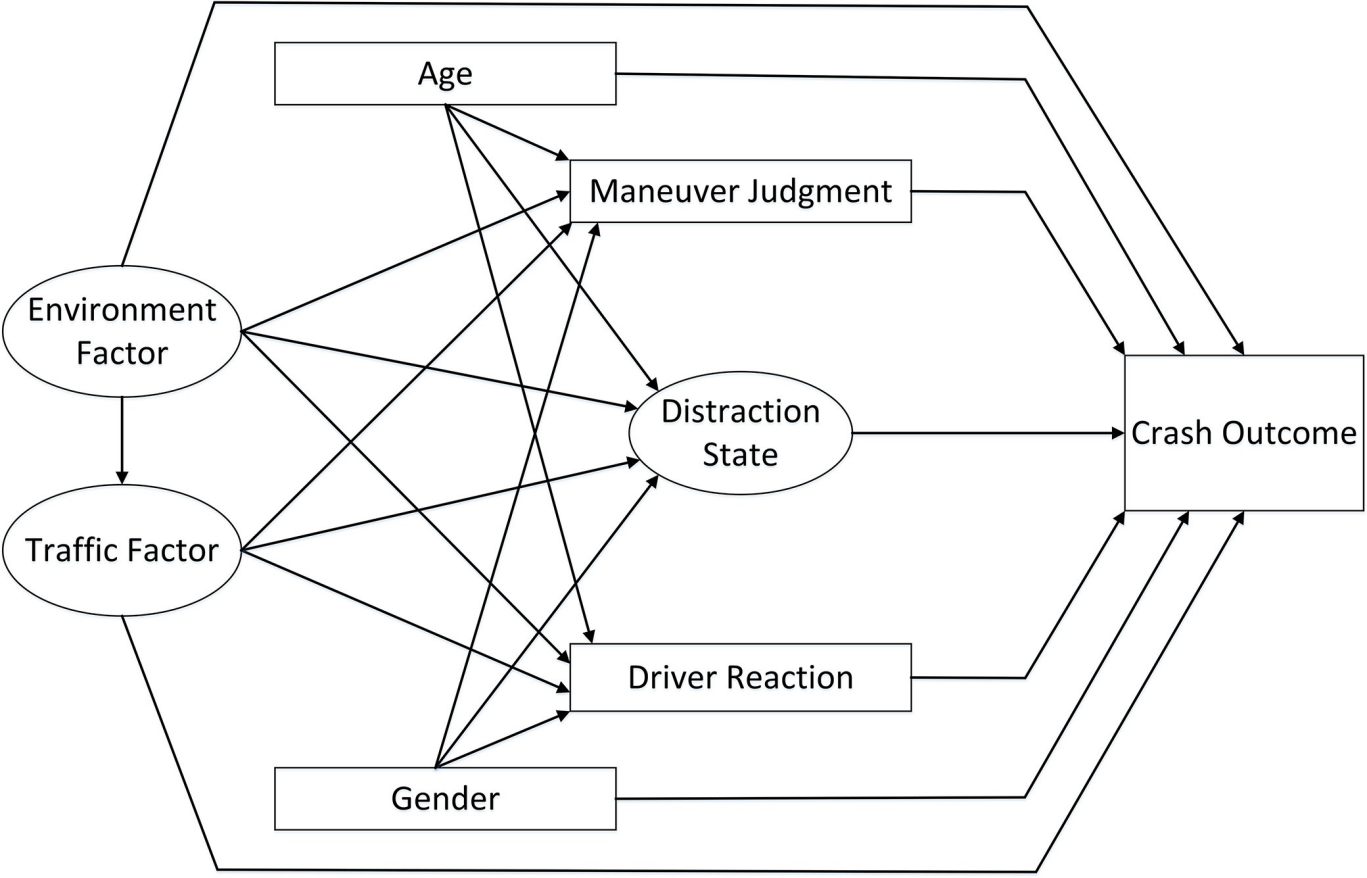
Structural equation initial influence path.

There are two primary approaches to solving SEM models: Covariance-based SEM (CB-SEM) and Partial Least Squares SEM (PLS-SEM), which is based on variance analysis[22]. When compared to traditional CB-SEM, PLS-SEM offers several advantages. These advantages include not making assumptions about data distribution, having no restrictions on the number of observed indicators for each latent variable, not requiring observation independence, and being capable of estimating complex models even with small sample sizes [29]. Due to the frequent use of naturalistic driving data as the research subject in the field of traffic safety, it is often challenging to ensure that the data characteristics meet the normality requirements. Therefore, the application of PLS-SEM in the field of traffic safety has been gradually increasing [22, 25, 30]. In non-experimental research data, the moderating influence of influencing factors is typically small [31, 32]. Therefore, this study will focus on analyzing the direct effects and mediating roles of each influencing factor on event outcomes using the PLS-SEM. In this research, age, gender, distraction state, maneuver judgment, driver reaction, environmental factors, and traffic conditions serve as independent variables, with crash outcomes as the dependent variable. Additionally, traffic factors, maneuver judgment, distraction state, and driver reaction will be used as mediator variables to calculate the indirect effects of environmental factors, age, and gender on event outcomes. The potential influence pathways of each variable are depicted in **Fig 1**.

## 4. Results

### 4.1 Results of K-means clustering

**Fig 2:**
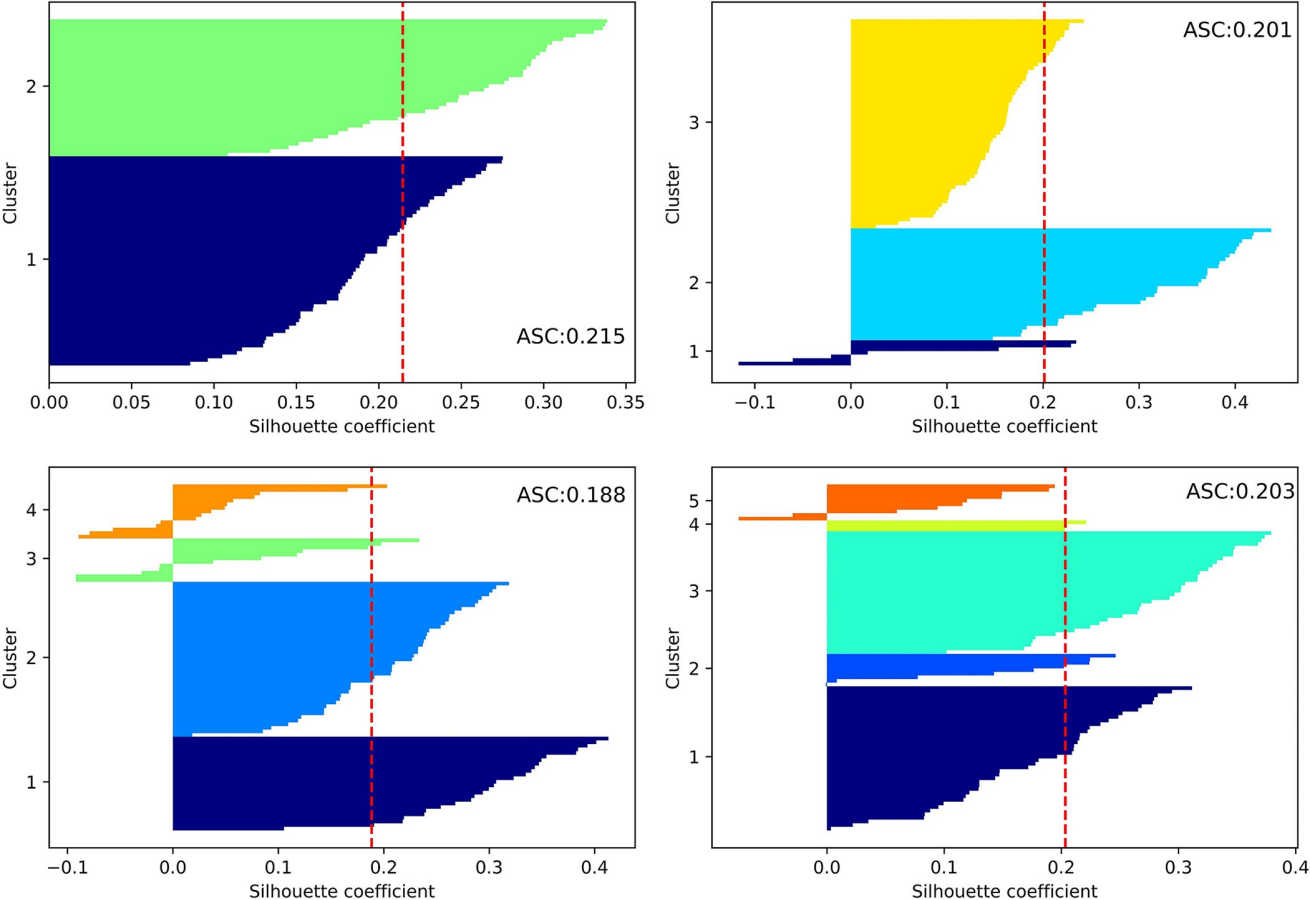
SC graph for different numbers of clusters.

**Fig 2** shows the SC for different values of k. As can be seen from the figure, only for k = 2, there is no negative SC; the average SC is higher than the other k cases, and the number ratios of the two clusters of drivers are also balanced. The above results show that the best clustering effect was achieved when k = 2.

This study used a K-means clustering method to classify 96 drivers into two risk-level clusters, with 51 drivers in cluster 1 and 45 in cluster 2. This study uses analysis of variance (ANOVA) to further explore the differences in drivers’ characteristics in risk-level clusters. **Table 4** reports the results of the comparison of the differences in each characteristic between the two clusters. From **Table 4**, it can be seen that 13 of the 22 clustering indicators significantly affected the clustering results.

**Table 4:** Comparison of differences in driving cluster characteristics.

Variables	Cluster 1(n = 51)		Cluster 2(n = 45)		F	P-value
	Mean	SD	Mean	SD		
**Gender**	0.530	0.504	0.698	0.457	3.082	0.082
**Age**	0.227	0.262	0.504	0.294	22.911	**0.000** **
**Crash ratio**	0.158	0.232	0.072	0.04	0.214	**0.024** *
**Maneuver judgment**						
Safe and legal event ratio	0.857	0.154	0.921	0.224	2.323	0.131
Safe but illegal event ratio	0.012	0.032	0.011	0.046	0.307	0.581
Unsafe but legal event ratio	0.074	0.107	0.012	0.045	9.455	**0.003** **
Unsafe and illegal event ratio	0.057	0.113	0.056	0.212	0.009	0.994
**Event nature**						
Single-vehicle event ratio	0.157	0.192	0.043	0.092	12.09	**0.001** **
Straight-line event ratio	0.602	0.234	0.651	0.344	0.753	0.388
Direction change event ratio	0.241	0.225	0.306	0.329	1.046	0.309
**Pre-incident maneuver**						
Steady driving event ratio	0.254	0.182	0.565	0.302	37.931	**0.000** **
Direction change event ratio	0.259	0.176	0.078	0.133	31.927	**0.000** **
Velocity change event ratio	0.487	0.229	0.357	0.304	6.121	**0.015** *
**Driver reaction**						
Braking reaction ratio	0.707	0.243	0.944	0.112	32.655	**0.000** **
Steering reaction ratio	0.121	0.176	0.021	0.072	13.37	**0.000** **
Accelerated reaction ratio	0.018	0.052	0.009	0.031	1.799	0.183
No reaction ratio	0.154	0.254	0.026	0.195	9.194	**0.003** **
**Distraction state**						
No-risk distraction event ratio	0.605	0.218	0.551	0.363	1.483	0.226
Low-risk distraction event ratio	0.240	0.215	0.385	0.342	4.624	**0.034** *
Mid-risk distraction event ratio	0.124	0.193	0.042	0.091	7.204	**0.009** **
High-risk distraction event ratio	0.031	0.054	0.022	0.083	0.489	0.486
**Multiple distractions event ratio**	0.204	0.172	0.092	0.184	9.478	**0.003** **

Note

*: p<0.05

**: p<0.01

### 4.2 Results of factor analysis

The initial latent variables were determined based on a summary of previous research and practical experience. To ensure the goodness of fit of the structural equations, factor analysis methods were required to test the fitness of the relevant observed and latent variables. **Table 5** reports the factor analysis results obtained with varimax rotation, a standard method that attempts to minimize factor complexity by making the loadings within each factor significantly different. Five factors are extracted for the relevant obvious variables based on the criterion of eigenvalues more significant than 1. The cumulative variance explained by the factors is 76.50% for cluster 1 and 74.48% for cluster 2. Based on the magnitude of the factor loading coefficient, it can be seen that factor 1 reflects the distraction state of the driver, where all three variables related to the distracted state have high loadings. Factors 2 and 3 reflect the traffic state, and all four variables associated with the traffic state have high factor loadings. Factors 4 and 5 reflect weather and lighting, respectively, with weather and lighting forming different factors with loading coefficients higher than 0.9. Therefore, weather and lighting cannot be combined to constitute latent variables of environmental factors. Instead, weather and lighting must be treated as independent endogenous variables to analyze the relationship of influence with other exogenous variables.

**Table 5:** Results of varimax rotation factor analysis.

Variable	Factor 1	Factor 2	Factor 3	Factor 4	Factor 5
**Cluster 1**					
Multiple distractions	**0.851**	0.086	0.029	-0.086	-0.02
Distraction behavior 1	**0.721**	0.06	-0.097	-0.125	-0.146
Distraction behavior 2	**0.787**	-0.079	0.075	0.17	0.052
Traffic control	0.018	**0.889**	0.044	0.03	-0.041
Relation to junction	0.06	**0.866**	0.116	0.021	0.036
Traffic density	0.04	-0.073	**0.906**	0.039	0.043
Locality	-0.064	0.445	**0.598**	-0.12	-0.136
Lighting	-0.042	0.035	-0.025	**0.974**	-0.062
Weather	-0.086	-0.016	-0.028	-0.063	**0.978**
**Cluster 2**					
Multiple distractions	**0.816**	-0.12	0.014	-0.148	0.129
Distraction behavior 1	**0.717**	0.106	-0.258	-0.019	-0.329
Distraction behavior 2	**0.767**	-0.041	0.137	0.159	0.205
Traffic control	0.006	**0.889**	0.061	-0.047	-0.011
Relation to junction	-0.084	**0.769**	0.259	-0.086	0.042
Traffic density	-0.048	0.112	**0.843**	-0.089	-0.005
Locality	0.068	0.378	**0.597**	0.114	-0.237
Lighting	-0.016	-0.017	-0.028	**0.976**	0.054
Weather	0.111	0.05	-0.131	0.057	**0.914**

Factor analysis results were used as a reference to correct the initial structural equation paths according to the actual significance of the observed variables. The four traffic-related observed variables in factors 2 and 3 were combined into a latent traffic factor. The environmental factors in the original path were divided into different weather and lighting variables.

### 4.3 Results of the SEM model

The modified structural equation assesses the relationship between the influencing factors and crash outcomes for the two driver clusters in the risk scenario. Commonly used fit evaluation metrics include *χ*^2^/*df*, root mean square error of approximation (RMSEA), comparative fit index (CFI), goodness of fit index (GFI), and normed fit index (NFI). From **Table 6**, it can be seen that except for the NFI, which is slightly lower than 0.9, all other indicators meet the test requirements, and the goodness of fit of the structural models for both driver clusters can be considered to be acceptable.

**Table 6:** Fit statistics for SEM.

Fit index	Criteria of acceptable fit	Cluster 1	Cluster 2
*χ*^2^/*df*	<3	2.647	2.993
RMSEA	<0.08	0.053	0.056
CFI	>0.9	0.923	0.901
GFI	>0.9	0.964	0.946
NFI	>0.9	0.889	0.876

#### 4.3.1 Direct effect

**Fig 3:**
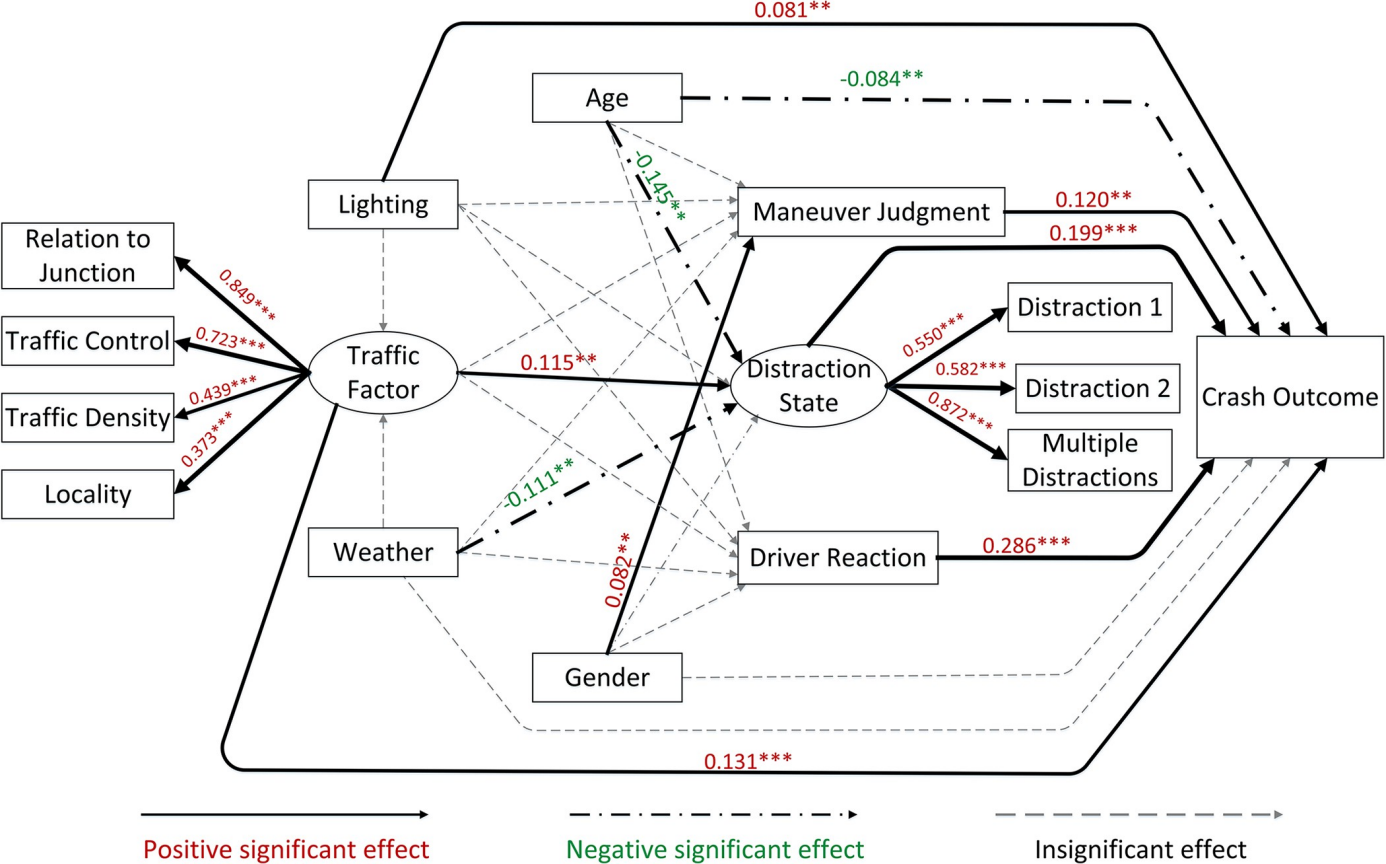
SEM results for cluster 1.

**Fig 4:**
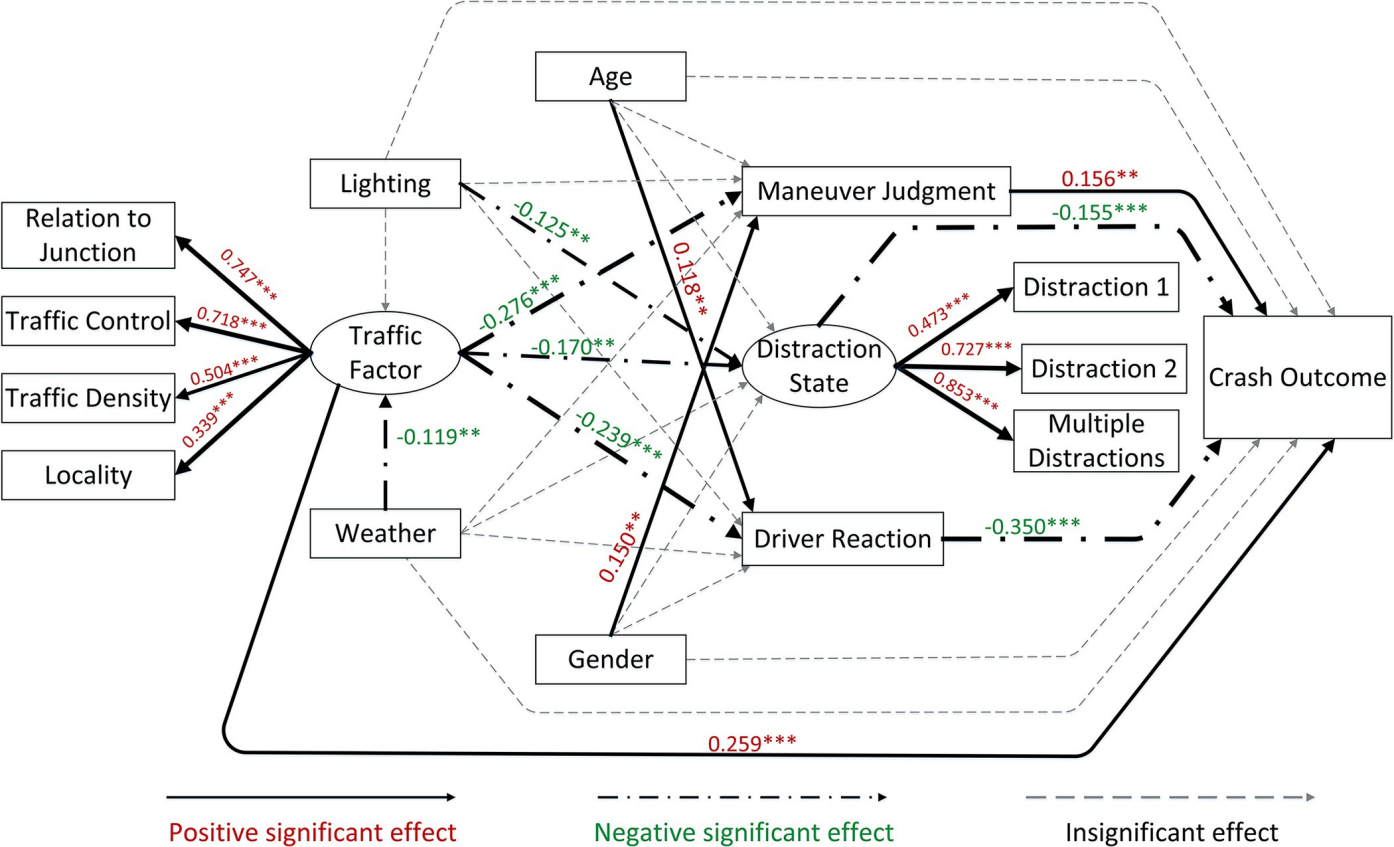
SEM results for cluster 2.

**Figs 3** and **4** show the standardized regression weights for the two driver clusters. To better visualize the model results, solid lines reflect positive significant influence relationships, chain dotted lines represent negative significant influence relationships, and dashed lines represent non-significant paths. Path label values showing the corresponding factor loadings and linewidths are correlated with factor loading. The factor loading represents the standardized strength of the relationship between variables, with positive and negative values representing positive and negative correlations, respectively. To illustrate using the direct influence path of the crash outcome as an example: given that the near-crash code in this study is represented by 0 and the crash code by 1, when the factor loading is positive, it represents that the probability of the vehicle collision will increase when this influence factor variable increases. The following conclusions can be drawn from the models.

*1*. *Crash outcome*. Six factors in driver cluster 1 significantly influenced crash outcomes. Among them, driver reaction (β = 0.286, P < 0.001) and driver distracted state (β = 0.199, P < 0.001) were the characteristics with larger factor loadings. Traffic factors (β = 0.131, P < 0.001) and maneuver judgment (β = 0.120, P < 0.001) had similar influence loadings, while light had a smaller influence loading (β = 0.081, P < 0.033). The five indicators had positive and significant effects. This indicates that cluster 1 drivers are vulnerable to crashes when they adopt a non-braking reaction in risk scenarios with poor traffic factors and lighting conditions, high distraction severity, and unsafe maneuver judgments. Age (β = -0.084, P = 0.032) significantly and negatively affected the crash outcome in this cluster, indicating that younger drivers in cluster 1 are more likely to be involved in crashes in a risk scenario. The two characteristics of driver reaction (β = -0.350, P < 0.001) and distraction (β = -0.155, P < 0.001) for cluster 2 showed an opposite influence relationship to that of cluster 1. Moreover, for cluster 2 drivers, the traffic factor (β = 0.259, P < 0.001) had a significantly higher degree of influence, and age and light conditions did not directly affect the crash outcome.

*2*. *Distraction state*. The observed variables that constituted the driver’s distracted state were all significant influencing variables, with the most significant effect of the multiple distraction variable (cluster 1: β = 0.872, P<0.001; cluster 2: β = 0.853, P < 0.001). Moreover, for driver cluster 1, age (β = -0.145, P = 0.003), traffic factors (β = 0.115, P = 0.024), and weather (β = -0.111, P = 0.018) also had a significant effect on driver distraction. However, for cluster 2 drivers, age and weather did not significantly affect distraction; light conditions became a significant influencing variable (β = -0.125, P = 0.011). The effect of traffic factors on driver distraction status was opposite in the two clusters (cluster 1: β = 0.115, P = 0.024; cluster 2: β = -0.170, P = 0.004).

*3*. *Driver reaction and maneuver judgment*. Cluster 1 drivers demonstrated only one indicator of gender (β = 0.082, P = 0.049) that was significantly correlated with drivers’ maneuver judgment, and male drivers were more likely to perform unsafe driving maneuvers in the event of risk scenarios. For cluster 2 drivers, gender (β = 0.150, P = 0.003) and traffic factors (β = -0.276, P < 0.001) both significantly influenced driver maneuvers, and age (β = 0.118, P = 0.023) and traffic factors (β = -0.239, P < 0.001) both significantly influenced driver crash avoidance reactions.

*4*. *Traffic factors*. Among the four observed variables that constitute traffic factors, whether the vehicle travels at an intersection is the most significant influencing traffic factor (cluster 1: β = 0.849, P < 0.001; cluster 2: β = 0.747, P < 0.001), and the road location at which the vehicle travels are less influential (cluster 1: β = 0.373, P < 0.001; cluster 2: β = 0.339, P < 0.001). The results of the latent inter-variate effects show that traffic factors are not significantly associated with environmental factors in the event of the risk scenarios in Cluster 1. In contrast, the CNC data for cluster 2 showed a negative effect of weather on traffic (β = -0.119, P = 0.045).

#### 4.3.2 Mediation effect

From the above mentioned SEM results, it can be observed that indirect significant relationships exist between numerous variables. This study uses the bootstrap method to test whether the variables affect crash outcomes through their mediating effects. This method has accurate probabilistic computational performance and has been recommended by several researchers for studies assessing such indirect effects [33]. The Bootstrap method generates bias-corrected confidence intervals, taking the 2.5th percentile and 97.5th percentile values of the effect parameter distributions as the upper and lower bounds, and checking whether the 95% CIs contain 0; if the intervals do not contain 0, it indicates that the mediating effect is significant, otherwise it is not significant.

**Table 7** reports all significant mediational paths for both driver clusters. The table shows that driver cluster 1 has multiple mediating effects, with lighting, traffic factors, and age as partial mediating influence variables and weather and gender as full mediating influence variables. In particular, distraction is associated with multiple factors. Each variable with a mediating effect affects the crash outcome by affecting the driver’s distraction state. Notably, cluster 1 drivers’ gender shows two opposite effects when distraction (β = -0.017, P = 0.037) and maneuver judgment (β = 0.010, P = 0.048) are used as mediators. Because of the more significant effect of distraction as a mediator, the gender should indicate a negative effect relationship on crash outcomes, demonstrating that male drivers are more likely to successfully crash avoidance in risk scenarios.

**Table 7:** Results for the mediation effect.

Path	Estimate	S.E.	Bias-corrected 95%CI			Decision
			Lower	Upper	P-Value	
**Cluster 1**						
LIG→CO	0.081	0.039	0.002	0.156	0.04	Partial mediation
LIG→DS→CO	-0.017	0.011	-0.047	-0.002	0.021	
TF→CO	0.131	0.046	0.039	0.218	0.007	Partial mediation
TF→DS→CO	0.023	0.013	0.004	0.057	0.019	
AGE→CO	-0.084	0.035	-0.148	-0.014	0.018	Partial mediation
AGE→DS→CO	-0.029	0.017	-0.075	-0.006	0.004	
WEA→DS→CO	-0.022	0.012	-0.053	-0.005	0.005	Full mediation
GEN→MJ→CO	0.010	0.007	0.001	0.028	0.048	Full mediation
GEN→DS→CO	-0.017	0.012	-0.049	-0.001	0.037	Full mediation
**Cluster 2**						
WEA→DR→CO	-0.021	0.013	-0.055	-0.001	0.035	Full mediation

Note: LIG: Lighting; CO: Crash Outcome; DS: Distraction State; TF: Traffic Factor; WEA: Weather; AGE: Age; GEN: Gender; MJ: Maneuver Judgment; DR: Driver Reaction.

In contrast, among all influencing factors, only the weather variable had a mediating effect on the crash results for driver cluster 2. The model results show that drivers in cluster 2 have no braking reaction in poorer weather, but this increases the likelihood of successful crash avoidance.

## 5. Discussion

### 5.1 Analysis of K-means clustering results

The clustering results show that the 96 drivers can be divided into two homogeneous clusters, with 53% and 47% of drivers in the two clusters. Cluster 1 drivers had a higher average crash ratio than cluster 2 drivers. Cluster 1 drivers had an average crash ratio of 15.8%, while cluster 2 drivers had an average crash ratio of 7.2%. Therefore, in terms of the average crash ratio, driver cluster 1 is more crash-prone than driver cluster 2.

Analysis of age characteristics showed a higher percentage of younger drivers in cluster 1. Previous studies have shown that young drivers tend to be characterized by severely distracted driving, weak safety awareness, inexperienced driving, lack of driving skills, and poor risk perception [34, 35].

The clustering results in this study are consistent with those of existing studies that assess the driving characteristics of young drivers. Cluster 1 drivers exhibited a higher incidence of moderate- and high-risk distractions and multiple distractions, indicating a significantly higher severity of distraction than cluster 2 drivers. In addition, Cluster 1 drivers also showed a higher percentage of unsafe driving maneuver judgment characteristics. This driving performance is significantly associated with the occurrence of crashes. For instance, Liang and Yang [36] identified that distraction is a significant factor in crashes and that more than 30% of traffic fatalities and injuries are associated with distraction [37]. Song [38] confirmed that the frequency of unsafe maneuvers is an essential risk assessment indicator and that drivers with a higher cumulative violation frequency are more likely to be involved in crashes.

A comparison of the crash avoidance reactions of the two driver clusters in risk scenarios shows that cluster 1 drivers exhibit a more hazardous crash avoidance maneuver. They have a lower frequency of brake reactions than cluster 2 drivers and a higher frequency of no-reaction maneuvers than cluster 2 drivers. This characteristic reflects the poor driving skills of cluster 1 drivers and the need to improve crash avoidance skills. A comparison of the pre-incident maneuvers for the two driving clusters shows that cluster 2 drivers have a much lower probability of CNCs during velocity and direction changes than cluster 1 drivers. This also proves that cluster 2 drivers are more skilled in vehicle control skills and can make safer maneuvers to avoid crashes when the vehicle undergoes lateral or longitudinal state changes. A study based on naturalistic driving data proved that the correct judgment of current risk scenarios and timely crash avoidance maneuvers can significantly avoid accidents. Poor driving skill is a critical cause of crashes [29]. Thus, it can be seen that cluster 1 drivers are more likely to be involved in crashes than cluster 2 drivers.

A comparison of the nature of CNC events shows a significant difference between cluster 1 and cluster 2 drivers in the single-vehicle event ratio. Cluster 1 drivers had an average single-vehicle event rate of 15.7%. In contrast, cluster 2 drivers had an average single-vehicle event rate of only 4.3%, indicating that cluster 1 drivers are frequently involved in crashes with non-vehicle objects. It has long been shown that single-vehicle crashes, particularly vehicle-object crashes, occur with a high probability in clusters of young drivers with limited driving experience [39]. It has also been noted that drivers with weaker driving skills have difficulty performing correct avoidance maneuvers in the face of unexpected events, such as sudden pedestrian crossings, resulting in an increased probability of collision with pedestrians. It can be seen that the nature of the CNCs also demonstrates a significant difference in the level of driving skills between the two clusters.

Finally, a summary analysis of the driver characteristics of the two clusters was presented. A high proportion of young drivers, severely distracted driving, weak safety awareness, inexperienced driving, and lack of driving skills characterize cluster 1 drivers. The clustering results demonstrate that the cluster has a high crash ratio; therefore, it can be defined as a crash-prone high-risk driving cluster. Cluster 2 drivers exhibit the opposite driving performance to cluster 1 drivers and have a lower crash ratio, thus defining cluster 2 as a low-risk driving cluster.

### 5.2 Analysis of structural equation results

#### 5.2.1 Driving performance

The model results show that three characteristics that reflect driving performance—distraction, maneuver judgment, and driver reaction—are directly related to crash outcomes in risk scenarios. Among them, the maneuvering judgment characteristics of both driving clusters have similar positive factor loadings, indicating that performing hazardous driving behaviors will increase the likelihood of crash outcomes regardless of the cluster. This relationship is logical, and the association between typical risk-driving behaviors, such as speeding, following too closely, illegal lane-changing, and crash outcomes has long been proven [40]. The World Health Organization (WHO) suggests that speed is directly linked to crashes. For every 1 km/h increase in the average vehicle speed, there is a 4% increase in crashes resulting in injuries and a 3% increase in fatal crashes. It has also been identified that close following causes drivers to have an insufficient reaction time for braking response, which is a crucial cause of rear-end accidents [41, 42]. In addition, illegal lane changes, such as the non-use of turn signals before changing lanes and crossing solid lane markings, have also been demonstrated to be causal factors in crashes [43].

Notably, the distraction and driver reaction characteristics exhibited opposite effects between the two driver clusters. For high-risk driver clusters 1, severe distracted states and non-braking crash avoidance responses increase the probability of vehicle crashes, a general conclusion now generally accepted by research [36, 37]. For low-risk driving cluster 2, the model results suggest that increased distraction levels and non-braking maneuvers reduce the probability of driver crashes, which is inconsistent with the mainstream findings. Further studies are required to determine the causes of these findings.

Further analysis of the distraction characteristics revealed that cluster 2 drivers habitually observed the road conditions through the left and right windows. These actions of watching the traffic through the car windows were judged as distractions in the 100-car study. However, it has been noted that observing road conditions can help drivers gain a comprehensive understanding of the surrounding traffic environment and is essential for safe driving [44, 45]. The habit of observing road conditions must be developed through extensive driving experience, and novice teenage drivers may almost focus exclusively on the road ahead [46]. Older and more experienced drivers are observed in cluster 2. Although the distraction level is higher owing to the observation of road conditions, the comprehensive mastery of road conditions improves the success rate of crash avoidance for drivers in this cluster.

Analysis of driver reaction characteristics shows that cluster 1 drivers tend to be in a no-reaction state when braking is not performed, missing the optimal time to avoid a collision. However, cluster 2 drivers are experienced with excellent hazard judgment and often choose steering maneuvers for timely collision avoidance based on an accurate judgment of risk scenarios. Existing studies have demonstrated that non-braking maneuvers provide better collision avoidance in some risk scenarios [47]. For instance, steering avoidance requires a shorter longitudinal distance than braking avoidance at higher driving speeds and lower adhesion coefficients, thus providing higher crash avoidance effectiveness [48].

#### 5.2.2 Demographic characteristics

The relationship between the demographic characteristics and crash outcomes differed significantly between the two driver clusters. For high-risk driver cluster 1, age directly affects event outcomes, manifested by a decrease in the likelihood of collisions in risky scenarios as driver age increases within this cluster. Additionally, the age characteristics of this cluster indirectly impact event outcomes through the mediating factor of distraction state. Older drivers in this cluster tend to reduce distracted driving behaviors, thus decreasing the likelihood of collisions. The distraction characteristics of drivers in cluster 1 have the same impact on both direct and indirect effects. This conclusion is consistent with the results obtained from clustering and is confirmed by existing studies [34, 35, 49]. In driver cluster 1, gender does not have a direct and significant impact on event outcomes. Instead, it exerts an indirect influence on event outcomes by affecting maneuver judgment and distraction characteristics. When maneuver judgment is considered as a mediating factor, the influence of gender in this cluster becomes apparent as follows: males tend to be more prone to engaging in risky driving behaviors, ultimately leading to an increased likelihood of collisions indirectly. This inclination may be associated with the impulsive and adventurous traits often attributed to young males. Available studies show that men report significantly more risk-driving behaviors than women and that anger, impulsivity, and risk driving are common among young male drivers [50]. However, when distraction characteristics are considered as a mediating factor, the impact of gender on event outcomes in this driving cluster takes on a contrasting pattern. It appears that males are less inclined to engage in distracted driving behaviors, which consequently reduces the probability of vehicle collisions.

For cluster 2 drivers, gender and age characteristics did not significantly affect crash outcomes. This conclusion is justified by the fact that the drivers in this cluster are already skilled and experienced. Therefore, drivers no longer experience significant changes in driving skill levels as they age. Second, related studies comparing historical driving data for young drivers and experienced drivers also indicate that gender is no longer a significant factor in crash outcomes in experienced driver clusters [51, 52].

#### 5.2.3 Environmental and traffic factors

The model results indicate that traffic factors are crucially significant for both driver clusters. Drivers are vulnerable to crashes on complex urban roads with intersections, high traffic density, and the presence of traffic control. Environmental factors have a much smaller direct effect on event outcomes than traffic factors. The model results indicated that weather conditions did not directly influence crash outcomes in either driver cluster. Lighting has a direct effect only on cluster 1 drivers, who are prone to crash events when the illumination is poor. This phenomenon can be attributed to several reasons. First, the most direct effect of poor lighting is visibility reduction [53]. Reduced visibility leads to the weakened road observation ability of inexperienced drivers and the inability to rapidly detect risk scenarios. Second, poorly lit environments are prone to driving fatigue, leading to driving errors that can lead to crashes [54].

There are several mediating effects stemming from environmental and traffic factors. Most of these mediating pathways indicate that when environmental or traffic conditions are less favorable, these factors assist both categories of drivers in reducing the probability of collision accidents. This assistance is achieved through the reduction of distracted behaviors and the promotion of appropriate driving responses. This is due to fewer traffic disruptions and better weather conditions, making it easy for drivers to let their guard down and unconsciously distract driving, speeding, and other unsafe driving performances. Poorer driving environment factors will help drivers improve their driving focus and caution, making it easier to complete timely crash avoidance maneuvers in risk scenarios. However, for cluster 1 drivers with limited experience, the indirect impact of traffic factors yields a contrasting effect. In complex traffic environments, they must exert significant effort to pay attention to traffic signals, pedestrians crossing the road, and nearby vehicles. This heightened demand on their attention leads to greater distraction and an increased susceptibility to collision accidents [55–57].

A comparative summary of the differential effects of collision-related factors on the two clusters of drivers reveals the primary distinctions in the impact of these factors: (1) Drivers in cluster 1 exhibit a higher propensity for engaging in risky distracted behaviors compared to those in cluster 2 and encounter challenges in making timely and accurate crash avoidance responses in risk scenarios. Further insight into the differential effects of the aforementioned influencing factors reveals that the conclusions regarding driving performance directly reflect the varying levels of driving skill between the two driver clusters. Drivers in cluster 1 exhibit less proficiency in driving skills compared to those in cluster 2. (2) Drivers in cluster 1 are significantly influenced by demographic characteristics, which not only have direct effects but also impact event outcomes through their influence on driving performance. In contrast, event outcomes for drivers in cluster 2 are no longer influenced by demographic characteristics. Drivers in cluster 1 demonstrate a phenomenon where their driving skills gradually improve and collisions decrease as they age. This indirectly underscores the differences in driving skill levels between the two driver cluster s. Hence, it can be concluded that the core difference between drivers in cluster 1 and cluster 2 lies in the mastery of driving skills. Combining the differences in the effects of crash influencing factors and the characteristics of cluster analysis between the two driver clusters, from a regulator’s perspective, we can identify the high-risk characteristics of drivers in cluster 1. They are more prone to causing vehicle collisions compared to drivers in cluster 2, and such drivers require targeted safety supervision measures to help prevent traffic accidents

## 6. Conclusions

This study presents an exploratory study to classify homogeneous driver clusters based on naturalistic driving data. Cluster analysis was used to identify homogeneous driver clusters at both risk levels. The two clusters differed significantly in terms of demographic characteristics, driving characteristics, and crash ratios. Further, the study explores the direct and indirect effects of each factor on the event outcome under the risk scenario for two driver clusters. The main findings are summarized as follows.

1. Regardless of the driver cluster, traffic factors and maneuver judgments positively affect crash outcomes. Poor traffic conditions and engagement in risk-driving behavior increases the likelihood of crashes in risk scenarios.
2. Some factors had opposite effects on the crash outcomes of the two clusters. For instance, cluster 1 drivers cause crashes owing to distractions and non-braking reactions; however, cluster 2 drivers have increased crash avoidance success owing to these behaviors.
3. Demographic characteristics were significantly associated only with crash outcomes for cluster 1 drivers. Not only does age have a direct effect on crash outcomes, it also has an indirect effect mediated by distraction, both manifested in younger drivers’ vulnerability to crashes. Gender had the opposite effect when mediated by distracting or maneuvering judgments. Gender had the most significant effect load when mediated by distraction. Therefore, it can be assumed that male drivers in this cluster were more likely to successfully avoid crashes in risk scenarios.
4. Most mediating effects of the environmental and traffic factors suggest that a poorer driving environment will help both driver clusters adopt safe driving behaviors. However, for cluster 1 drivers, complex road conditions increase the driving burden of young drivers and can easily lead to crashes.
5. The effect loading of the mediating effect is small; therefore, the traffic factors and lighting conditions for driver cluster 1 will be dominated by the direct effect, as shown by the worse the traffic environment and lighting conditions, the higher the probability of a crash. The weather factor fully mediates in both clusters; therefore, poorer weather reduces the likelihood of collision occurrence in the risk scenario.

Understanding the differences in the impacts between heterogeneous clusters can help develop effective road safety intervention policies. For cluster 1 drivers, inexperience and risk driving may be the leading causes of crashes in risky situations. Watching safety education videos, reducing young drivers’ danger and adventurous mentality, and conducting simulator training to develop crash avoidance skills is an effective way to improve safety. For cluster 2 drivers, whose existing driving skills can handle most unexpected situations, their safety awareness is crucial. Extensive safety campaigns and strict traffic safety policies can improve driver safety awareness, making cluster 2 drivers less likely to be involved in crashes.

These safety measures aim to improve drivers’ safety awareness and driving skills to avoid crashes. However, human causes can often be eliminated through vehicle and/or environmental changes [58]. First, existing driver assistance systems can be leveraged to help drivers reduce their driving risk. Requiring drivers to drive vehicles using automatic emergency braking would significantly improve driving safety. In addition, for clusters of high-risk drivers with poor driving skills, a speed limit can be imposed by the speed control system, and the following distance can be continuously controlled using the adaptive cruise control system. Studies have demonstrated that this method can reduce crashes under congested traffic conditions by 12–27% [41]. Second, the traffic environment can be improved by arranging traffic police officers to direct traffic in areas with poor traffic conditions and simplifying complex traffic signs at intersections to reduce the identification burden on drivers, thus avoiding crashes.

Although this study analyzed the mechanism of crash-influencing factors acting in risk driving scenarios and reached objective and reliable conclusions, there are still gaps and directions for further research.

First, this study focused on crash effect factor analysis for the average driver. However, for professional drivers with significant safety responsibilities, crash causation analysis in risk scenarios is critical. The impact factors and mechanisms of crash events will be significantly different for professional and non-professional drivers owing to the purpose of driving, the number of driving hours, and the difference in individual driving miles. The next step of this research cluster will rely on a state-funded project to collect a large amount of driving data from professional drivers to study the mechanisms of the factors influencing urban bus and long-haul truck collisions. Second, for drivers, the influence of each factor may change over time or with the occurrence of a crash. In future studies, we will consider whether changes in the effects of influencing factors exhibit specific patterns in homogeneous clusters. In addition, the differences in the time-varying patterns of influencing factors between heterogeneous clusters of drivers and the causes of these differences will be further explored to provide drivers with more time-sensitive traffic safety countermeasures.
